# *Grifola frondosa* as a natural additive enhancing antioxidant activity and nutritional value in gluten-free bread

**DOI:** 10.1038/s41598-026-40938-0

**Published:** 2026-02-25

**Authors:** Zbigniew Kobus, Monika Krzywicka, Agata Blicharz-Kania, Alicja Bosacka, Anna Pecyna, Eva Ivanišová, Katarzyna Kozłowicz, Eva Kovačiková, Lidia Ślusarczyk, Arkadiusz Matwijczuk, Marta Krajewska

**Affiliations:** 1 Department of Technology Fundamentals, University of Life Sciences in Lublin, Głeboka 28, Lublin, 20-612 Poland; 2 Department of Biological Bases of Food and Feed Technologies, University of Life Sciences in Lublin, Głeboka 28, Lublin, 20-612 Poland; 3 Institute of Food Sciences, Faculty of Biotechnology and Food Sciences, Slovak University of Agriculture, Trieda Andreja Hlinku 2, Nitra, 94976 Slovakia; 4 Food Incubator, AgroBioTech Research Centre, Slovak University of Agriculture, Trieda Andreja Hlinku 2, Nitra, 94976 Slovakia; 5 AgroBioTech Research Centre, Slovak University of Agriculture, Trieda Andreja Hlinku 2, Nitra, 94976 Slovakia; 6 Department of Biophysics, Faculty of Environmental Biology, University of Life Sciences in Lublin, Akademicka 13, Lublin, 20-950 Poland

**Keywords:** Biochemistry, Health care

## Abstract

Gluten-free breads enriched with maitake (*Grifola frondosa*) were developed to enhance functional value and evaluated for technological quality, antioxidant capacity, colour, texture, mineral composition, molecular features (FTIR), and consumer acceptance. Formulations containing 0–20% maitake (flour basis) were baked under standardized conditions. Compared with the control, the addition of maitake resulted in higher bread yield and moisture content, accompanied by a gradual reduction in specific volume as the level of supplementation increased. The enrichment also led to a significant improvement in nutritional quality, including higher protein and fat contents as well as enhanced levels of bioactive compounds (total phenolic content, total flavonoid content) and antioxidant capacity. Colour measurements indicated darker crumb colour with increasing levels of maitake, reflected by lower L* values, higher a* values, and largely unchanged b* and C* parameters. All enriched breads exhibited colour differences exceeding the perceptibility threshold (*ΔE* > 5). Texture analysis showed unchanged hardness up to 15% addition, with a significant increase at the highest level, while springiness decreased from 15%; cohesiveness and chewiness remained unaffected. Elemental analysis revealed increased mineral content, particularly potassium and zinc, which rose significantly from 5% addition and exceeded threefold levels at the highest supplementation. Spectroscopic analysis confirmed structural modifications within the bread matrix associated with interactions between mushroom components and the starch–protein network. Sensory evaluation revealed that breads containing low to moderate amounts of maitake were well accepted by consumers, whereas higher levels negatively affected flavour and texture. Overall, the results demonstrate that *Grifola frondosa* can be effectively used as a functional ingredient in gluten-free bread, enhancing its nutritional and antioxidant properties while maintaining acceptable technological and sensory quality, particularly at inclusion levels up to 5–10%.

## Introduction

Celiac disease is among the most common autoimmune disorders, with a prevalence of 0.5–1% in the general population, and its incidence has been rising in Western countries^1^. The only effective treatment is strict, lifelong adherence to a gluten-free diet^2^. Bread is a staple food consumed daily. A major challenge for food technologists is to replace gluten in breads with alternative ingredients while maintaining shelf life, texture, crumb structure, volume, and sensory quality^3^. Developing a suitable gluten substitute is difficult because gluten is the primary protein responsible for structure formation and for enhancing overall quality, elasticity, cohesiveness, and extensibility in wheat-based products^4^.

Rice flour is commonly used in gluten-free bread because of its low allergenic potential, neutral color, delicate flavor, and low cost^5^. The protein content of rice grain—similar to other cereals—is just under 10%^6^. An additional advantage of rice is its high content of digestible carbohydrates^7^. With higher proportions of rice flour, bread elasticity and cohesiveness increase, whereas chewiness, brittleness (crumbly texture), and hardness decrease^8^. Corn flour is also widely used in gluten-free breads. Corn provides storage protein (zein), vitamins, minerals, dietary fiber, and health-promoting compounds such as phenolics, anthocyanins, *β* -carotene, lutein, and flavonoids^9^. Potato starch is likewise widely used in the baking industry; its addition helps maintain bread freshness, imparts a pleasant flavor, and improves toasting properties^10^. Miedzianka et al.^11^ indicate that flours derived from oilseed by-products (evening primrose, milk thistle, and corn germ flours) can be important sources of total phenolics with strong antioxidant and antimicrobial activity.

In addition to core components such as gluten-free flours and starches, gluten-free bread formulations often include technologically functional and nutritional ingredients^3^. One approach to modifying gluten-free bread is the use of mushrooms, including medicinal species. Edible mushrooms provide carbohydrates, fiber, protein, essential amino acids, unsaturated fatty acids, vitamins, and minerals, while being low in energy density; consequently, they are regarded as healthy foods with favorable nutritional properties. Moreover, they contain bioactive compounds with beneficial health effects^12^.

Few studies have examined the effect of adding mushrooms to gluten-free breads^13–16^, and only Kobus et al.^17^ have investigated medicinal mushrooms. Sulieman et al.^13,14^ fortified gluten-free bread with *Agaricus bisporus* flour and inulin (3%, 6%, and 9%) and showed that these additions improved antioxidant, nutritional, and sensory properties, and reduced rheological moduli, which translates into lower product stiffness. Saw et al.^16^ added *Volvariella volvacea* (*V. volvacea*) to gluten-free bread and found increased protein and fiber contents with decreased fat content; the incorporation of *V. volvacea* also reduced bread hardness. According to Saccotelli et al.^15^, adding 15% button mushroom flour enhances functional properties without adversely affecting technological parameters. In their previous work, Kobus et al.^17^ demonstrated that gluten-free bread enriched with chaga flour (5%, 10%, 15%, 20%) had higher total phenolics, flavonoids, and antioxidant activity than the control bread; the mushroom addition also influenced textural properties and color. Based on consumer evaluations, up to 10% mushroom flour can be used in baking.

The effect of adding nutraceutical mushrooms to white wheat bread has been examined by several authors^18–20^. Seguchi et al.^18^ studied white wheat bread with maitake (0.017% – 0.140%) and observed decreased dough strength in Brabender farinography and deterioration of baking performance. Yuan et al.^19^ investigated the effect of *Auricularia auricula* (0% – 10%) on wheat bread and found increased viscosity and water-holding capacity alongside reduced dough stability and elastic modulus; incorporating more than 5% mushroom flour negatively affected loaf volume, height, texture, and appearance. Łysakowska et al.^20^ analyzed additions of 3%, 6%, 9%, and 12% Reishi (*Ganoderma lucidum*) and reported increased water absorption and extended dough stability time, decreased loaf volume, and higher moisture content. They also noted increases in total phenolics, antioxidant capacity, dietary fiber, protein, and essential minerals such as calcium, iron, and manganese. Based on their results, the authors recommend not exceeding 6% due to adverse effects on sensory quality.

An especially promising mushroom—with not only health-promoting properties but also desirable sensory attributes—is the hen-of-the-woods (*Grifola frondosa*), also known by its Japanese name, maitake. It is a mushroom of high nutritional and nutraceutical value^21^. The available literature indicates that *G. frondosa* exhibits anticancer, anti-inflammatory, antidiabetic, and antioxidant activities^21–23^. These effects are linked to its rich chemical composition. The principal bioactive constituents are *β* -glucans—particularly the so-called D-fraction, a *β* -glucan complex containing roughly 30% protein—along with fraction X, grifolan, fraction MZ, and MT-*α*-glucan^24,25^.

In this study, infrared spectra were recorded using Fourier-transform infrared (FTIR) spectroscopy, a widely applied analytical technique that enables rapid, non-destructive examination of composition and structural changes at the molecular level in food products. Owing to its high sensitivity, FTIR allows the identification of characteristic bands corresponding to proteins, polysaccharides, lipids, and water, thereby facilitating the analysis of complex products such as bread. In the context of adding maitake, this technique makes it possible to monitor interactions between its bioactive constituents and the bread matrix, and to assess the impact on protein, starch, and lipid structures. The application of FTIR thus provides critical insights into molecular alterations, supporting both qualitative assessments and evaluations of the functional properties of such products^26–29^.

To the best of our knowledge, there are no reports on fortifying gluten-free breads with maitake. Accordingly, the aim of this study is to enhance antioxidant properties and to analyze the quality, textural, color, and sensory parameters of gluten-free bread formulated with maitake mushrooms.

## Results and discussion

### Evaluation of bread quality attributes

Table 1 presents the results for specific volume, bread yield, total baking loss, and moisture of gluten-free bread enriched with maitake mushroom.

**Table 1:** Bread making characteristics.

Probe	Specific volume [cm3·g-1]	Bread yield [%]	Total baking loss [%]	Moisture [%]
**0**	1.33 ± 0.08^a^	171.57 ± 3.10^b^	16.71 ± 1.62^a^	49.38 ± 0.08^e^
**5**	1.32 ± 0.01^ab^	179.93 ± 1.36^a^	15.09 ± 0.81^a^	50.47 ± 0.16^d^
**10**	1.12 ± 0.02^abc^	180.71 ± 0.53^a^	15.28 ± 0.08^a^	52.13 ± 0.11^c^
**15**	1.07 ± 0.08^bc^	186.19 ± 1.18^a^	14.20 ± 0.54^a^	52.60 ± 0.14^b^
**20**	1.01 ± 0.09^c^	186.82 ± 2.21^a^	14.05 ± 1.10^a^	53.12 ± 0.01^a^

0 – control probe, 5 – gluten-free bread with the addition of 5% maitake mushroom; 10 – gluten-free bread with the addition of 10% maitake mushroom; 15 – gluten-free bread with the addition of 15% maitake mushroom; 20 – gluten-free bread with the addition of 20% maitake mushroom. Data value of each parameter with different superscript letter in rows are significantly different (Tukey test p *≤* 0.05).

Fortifying gluten-free bread with maitake led to a 24% decrease in specific volume, from 1.33 cm³·g^-1^ (control) to 1.01 cm³·g^-1^ (20% addition). Based on ANOVA and Tukey’s test results (*p* < 0.05), statistically significant differences were found between the control bread and the breads with 15% and 20% maitake. Kobus et al.^17^ likewise observed a significant 21% reduction in the specific volume of gluten-free bread with a 20% chaga addition. Ulziijargal et al.^30^ added 5 g of various mushrooms (*Antrodia camphorata*,* Agaricus blazei*,* Hericium erinaceus*,* Phellinus linteus mycelia*) to wheat breads and, in all cases, reported a decrease in specific volume. Losoya-Sifuentes et al.^31^ also noted a significant reduction in the specific volume of wheat bread after adding *Pleurotus ostreatus* (*P. ostreatus*) (0%, 5%, 10%, 15%, 20%), from 5.06 cm³·g^-1^(control) to 2.64 cm³·g^-1^(20% addition). Wahab et al.^32^ similarly reported a decrease in the specific volume of wheat bread with *P. ostreatus* at 0, 5%, and 10%. Sławińska et al.^33^ found that a 2.5% addition of white button mushrooms did not cause a meaningful change in this parameter, whereas increasing the level to 5% led to a decrease.

The addition of maitake increased bread yield by 9%, from 171.57% (control) to 186.82% (20% addition). Statistically significant differences were observed between the control and all breads containing the mushroom. Łysakowska et al.^20^ likewise reported a statistically significant 5% increase in bread yield after adding 12% reishi to wheat bread.

Total baking loss decreased by 16%, from 16.71% (control) to 14.05% (20% addition). According to the statistical analysis, no significant differences were detected among the breads tested. Łysakowska et al.^20^ also noted a reduction in total baking loss; with a 12% reishi addition, the decrease was 23%, and statistically significant differences were found for breads with 9% and 12% addition compared with the control.

Bread moisture increased by 8%, from 49.38% to 53.12%. ANOVA followed by a post hoc test indicated statistically significant differences between the control and all breads with maitake. Łysakowska et al.^20^ likewise observed increased moisture following a 12% reishi addition (10% increase); the difference was not significant only for the 3% level compared with the control, while breads with 6%, 9%, and 12% showed statistically significant increases. Losoya-Sifuentes et al.^31^ also reported a moisture increase (by 4%) in wheat bread with 20% *P. ostreatus*, although this change was not statistically significant.

### Chemical analysis

The results of chemical analysis in terms of bioactive substances, their antioxidant activity and protein and fat content are presented in Table 2.

**Table 2:** Chemical analysis of maitake-doped breads.

Probe	TPC [mg GAE· g _d. m_.^-1^]	TFC [mg QE· g _d. m_.^-1^]	DPPH [µg TE· g _d. m_.^-1^]	FRAP [µg TE· g _d. m_.^-1^]	Protein [%]	Fat [%]
0	0.59 ± 0.02^e^	0.09 ± 0.00^d^	3.41 ± 0.05^e^	1.96 ± 0.00^e^	7.18 ± 0.03^e^	5.40 ± 0.03^d^
5	1.09 ± 0.05^d^	0.11 ± 0.01^d^	3.97 ± 0.11^d^	2.44 ± 0.10^d^	8.21 ± 0.04^d^	5.46 ± 0.04^d^
10	1.42 ± 0.03^c^	0.16 ± 0.02^c^	4.62 ± 0.07^c^	2.97 ± 0.10^c^	8.92 ± 0.04^c^	5.74 ± 0.04^c^
15	1.77 ± 0.04^b^	0.21 ± 0.00^b^	5.01 ± 0.03^b^	3.55 ± 0.02^b^	9.90 ± 0.03^b^	6.39 ± 0.05^b^
20	2.26 ± 0.04^a^	0.31 ± 0.00^a^	5.68 ± 0.04^a^	4.16 ± 0.04^a^	10.85 ± 0.02^a^	6.62 ± 0.05^a^

0 – control probe; 5 – gluten-free bread with the addition of 5% maitake mushroom; 10 – gluten-free bread with the addition of 10% maitake mushroom; 15 – gluten-free bread with the addition of 15% maitake mushroom; 20– gluten-free bread with the addition of 20% maitake mushroom. Data value of each parameter with different superscript letter in rows are significantly different (Tukey test. p *≤* 0.05).

The incorporation of maitake mushroom into bread at levels of 5%, 10%, 15%, and 20% produced a significant (p *≤* 0.05) increase in both protein and fat contents. Relative to the control bread, protein content rose by approximately 51.1%, while fat content increased by about 22.6%. Proteins present in mushrooms are important bioactive constituents; their content varies widely—from 13.3 to 38.5 g·100 g^-^¹—depending primarily on the mushroom species^34^. The amino acid composition and sequences, as well as polypeptide chain length, can determine the specific biological activity of these compounds. As emphasized by Thatoi and Singdevsachan^35^, the nutritional quality of mushroom protein may even exceed that of milk, meat, or eggs. Mushroom proteins are characterized by a high content of essential amino acids and elevated levels of glutamic acid, aspartic acid, and arginine^36^.

Analysis of the antioxidant properties of maitake (on a dry-matter basis) showed that TPC was 10.60 ± 0.10 mg GAE·g_d.m_^-1^, TFC was 2.07 ± 0.041 mg QE·g_d.m_^-1^, DPPH was 18.37 ± 0.11 *µ*g TE·g_d.m_^-1^. and FRAP was 16.60 ± 1.17 *µ*g TE·g_d.m_^-1^. Fortifying gluten-free bread with maitake increased TPC by 283%, from 0.59 ± 0.02 (control) to 2.26 ± 0.04 mg GAE·g_d.m_^-1^.(20% addition). Based on ANOVA and Tukey’s test (*p* < 0.05), statistically significant differences were found among the breads. Maitake addition also raised TFC by 244%, from 0.09 ± 0.00 (control) to 0.31 ± 0.00 mg QE·g_d.m_^-1^.(20% addition). Statistical analysis indicated no significant difference in TFC between the control and the 5% addition. For DPPH, the value increased by 67%, from 3.41 ± 0.05 (control) to 5.68 ± 0.04 *µ*g TE·g_d.m_^-1^.(20% addition). For FRAP, an increase of 112% was recorded, from 1.96 ± 0.00 (control) to 4.16 ± 0.04 *µ*g TE·g_d.m_^-1^.(20% addition). According to ANOVA and Tukey’s test (*p* < 0.05), statistically significant differences in DPPH and FRAP were observed among the breads tested.

No studies were found in the available literature on the effect of maitake addition on TPC and TFC in bread. Therefore, the discussion cites works on fortifying bread with other mushroom species. Only Kobus et al.^17^, in their earlier study, analyzed the fortification of gluten-free bread with a medicinal mushroom and reported increases in TPC by 78% and TFC by 81% for breads with a 20% chaga addition compared with the control. Sulieman et al.^13^ examined gluten-free bread enriched with *Agaricus bisporus* flour and inulin at 0%, 3%, 6%, and 9% and observed that the addition of mushrooms increased phenolic content from 8.83 to 19.05 mg GAE·g_d.m_^-1^, compared with 7.79 mg GAE·g_d.m_^-1^. in the control. Saccotelli et al.^15^ also studied gluten-free bread, adding 15% mushroom flour (species not specified) and achieving TPC of 2.40 mg GAE·g_d.m_^-1^. and TFC of 0.45 mg QE·g_d.m_^-1^. Łysakowska et al.^20^ analyzed fortification of wheat bread with a medicinal mushroom; additions of 3%, 6%, 9%, and 12% reishi led to statistically significant increases in TPC and TFC compared with the control. TPC rose from 0.46 to 1.90 mg GAE·g_d.m_^-1^. No TFC was detected in the control bread, whereas with a 12% addition it reached 0.21 mg QE·g_d.m_^-1^. Zhang et al.^37^ investigated wheat bread with *Agaricus bisporus* powder (0–8%) and found that TPC increased from 28.34 to 64.16 mg GAE·100 g^-1^. Vlaic et al.^38^ enriched wheat bread with porcini powder (0%, 3%, 6%, 9%), resulting in an increase TPC from 39.88 to 64.46 mg GAE·100 g. In the study by Lu et al.^39^, additions of 5%, 10%, and 15% dried shiitake, sliced porcini, and fresh button mushrooms to wheat bread significantly increased TPC—from 4 to 11 mg GAE·g_d.m_^-1^. In Sławiska et al.^33^, bread enriched with 2.5 and 5% *Agaricus bisporus* powder showed TPC and TFC values ranging from 0.76 to 1.25 mg GAE/·g_d.m_.^−1^ for control and enriched samples, respectively.

In the literature, antioxidant activity data for DPPH and FRAP are reported in various units, hindering direct comparisons. Sulieman et al.^13^ observed increased DPPH activity with higher proportions of A. bisporus flour, with EC_50_ values ranging from 6.85 to 19.17 mg·mL^-1^. Saccotelli et al.^15^ reported that DPPH increased from 0.01% (control) to 4.55% inhibition after adding 15% mushroom flour to gluten-free bread. Kobus et al.^17^ indicated that adding the medicinal mushroom chaga increased DPPH and FRAP values for bread with a 20% addition by 238% and 199%, respectively, compared with the control. Łysakowska et al.^20^ also used a medicinal mushroom to fortify bread—wheat in this case—and likewise reported a significant increase in DPPH of more than 45%. Zhang et al.^37^ showed that DPPH activity in bread with mushroom powder rose from 18.77% (control) to 94.49% (8% addition). Vlaic et al.^38^ noted an increase in bread antioxidant activity from 13.91% to 21.91%. Lu et al.^39^, using different edible mushrooms, also found higher antioxidant activity in fortified breads—from 0.6 to 7.2 *µ*mol TE·g_d.m_^-1^. Similar results were obtained by Sławin´ska et al.^33^, where DPPH and FRAP increased with the amount of button mushroom powder, reaching 0.88–1.20 and 0.97–1.59 *µ*mol·g_d.m_^-1^, respectively.

### The colour of the bread

**Fig. 1:**
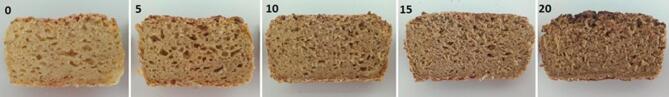
Gluten-free bread with maitake: 0%, 5%, 10%, 15%, 20% respectively (from left).

Colour is the key quality attribute influencing consumer preferences. Figure 1 presents images of bread. Table 3 reports the results of the colour analysis for gluten-free breads enriched with maitake mushroom alongside the control.

**Table 3:** Colour parameters obtained from the crumb of gluten-free bread with the addition of maitake.

Probe	L* - value	a* - value	b* - value	C* - value	∆E - value
0	55.12 ± 3.60^a^	1.87 ± 0.32^d^	24.69 ± 1.94^a^	24.76 ± 1.95^a^	-
5	47.21 ± 6.59^b^	4.56 ± 0.87^c^	24.08 ± 1.80^a^	24.51 ± 1.90^a^	8.38
10	46.10 ± 1.79^b^	5.83 ± 0.41^b^	24.77 ± 0.43^a^	25.45 ± 0.47^a^	9.85
15	44.55 ± 1.48^b^	6.52 ± 0.54^ab^	23.98 ± 1.22^a^	24.85 ± 1.30^a^	11.57
20	42.90 ± 2.11^b^	7.38 ± 0.81^a^	23.03 ± 4.08^a^	24.25 ± 3.65^a^	13.51

Probe	YI		WI		BI
0	61.72 ± 6.30^b^		50.62 ± 3.39^a^		23.52 ± 3.91^b^
5	74.04 ± 11.58^ab^		41.71 ± 6.00^b^		35.23 ± 10.41^ab^
10	76.53 ± 3.84^a^		40.51 ± 1.87^b^		37.62 ± 3.68^a^
15	77.01 ± 5.74^a^		39.23 ± 1.73^b^		39.07 ± 5.39^a^
20	76.71 ± 13.61^a^		37.88 ± 2.13^b^		40.78 ± 9.86^a^

0 – control probe; 5 – gluten-free bread with the addition of 5% maitake mushroom; 10 – gluten-free bread with the addition of 10% maitake mushroom; 15 – gluten-free bread with the addition of 15% maitake mushroom; 20– gluten-free bread with the addition of 20% maitake mushroom. Data value of each parameter with different superscript letter in rows are significantly different (Tukey test. p *≤* 0.05).

Incorporating maitake into gluten-free bread led to clear changes in color parameters. The highest lightness (L*) after baking was recorded for the control. Breads with maitake showed lower L* values, and higher maitake levels produced darker breads. The largest decrease was noted for the 20% addition (L* = 42.90; *−*22%), while the smallest decrease was observed for the 5% addition (L* = 47.21; *−*14%). Based on ANOVA followed by Tukey’s test, L* was significantly lower (*p* < 0.05) for all maitake-enriched breads versus the control, whereas no significant differences (*p* > 0.05) were detected among the 5%, 10%, 15%, and 20% levels.

The lowest a* value (redness) was recorded for the control (1.87). Maitake addition increased chromaticity toward red. Among the enriched breads, the lowest a* occurred at 5% (4.56; +74% vs. control), and the highest at 20% (7.38). ANOVA/Tukey indicated no significant differences (*p* > 0.05) in a between 10% and 15% or between 15% and 20%; all enriched breads differed significantly from the control (*p* < 0.05).

The lowest b* value (yellowness) was found for the 20% bread (23.03). However, ANOVA/Tukey showed no significant differences in b (*p* > 0.05) between the control and any maitake levels. Thus, the addition of maitake did not significantly affect yellowness in this study. All b* values were positive, indicating chromaticity shifted toward yellow.

Chroma (C*, color saturation) was lowest at 20% (24.25) and highest at 10%. Statistical analysis revealed no significant changes in *C* across treatments (*p* > 0.05).

The absolute color difference ∆*E* exceeded 5 for all breads containing maitake, indicating a clearly perceptible deviation from the control. The largest ∆*E* after baking was observed for the 20% bread.

The present results are consistent with earlier reports^13,37^ showing that mushroom-based additives—such as powdered button mushrooms or freeze-dried mushrooms—affect bread color. Supplementation typically lowered L* and increased a* and b*. For example, Sulieman et al.^13^ reported markedly lower crust and crumb L* in gluten-free breads with *Agaricus bisporus* polysaccharide flour and inulin, with the decrease proportional to the additive level; crumb color shifted from green toward red and both crust and crumb showed higher a* and b* than the control.

A comparable color response was observed here with maitake. Concordance with other studies—for instance, Zhang et al.^37^, who found that *A. bisporus* powder increases a* and decreases b*—supports the conclusion that the type and dose of mushroom additive are key determinants of bread color. Moreover, Eissa et al.^40^ showed that replacing even 15% of wheat flour with powdered legumes or mushrooms can beneficially influence product quality and nutritional value while altering L*, a*, b*, chroma, and browning in a dose- and material-dependent manner. The color changes in mushroom-enriched breads arise from the intrinsic color of the raw material and the synergistic effects of enzymatic browning, Maillard reactions, and caramelization^17,30,41,42^.

Incorporation of maitake into gluten-free bread produced a substantial increase in the yellowness index (YI), reaching the highest value (77.01) for bread with a 15% addition and the lowest for the control (61.72). Based on ANOVA and Tukey’s test (*p* > 0.05), no statistically significant differences in YI were found between the control bread and the bread with a 5% maitake addition. In turn, for the whiteness index (WI), a clear decrease was observed with increasing levels of maitake in the bread formulation. The highest WI (50.62) was found in the control sample, while the lowest value (37.88) was recorded for bread with a 20% maitake addition, indicating a pronounced darkening of the final product. Statistical analysis did not confirm significant changes in WI; between sample with addition of maitake.

Comparable relationships have been reported for other mushroom species, such as Lentinula edodes. Yen et al.^43^ showed that adding 5% chitin to bread dough reduced not only lightness but also the whiteness index, while increasing a* and b* values—evidence of intensified red and yellow hues. Likewise, Lin et al.^44^ confirmed that partially substituting wheat flour (5%) with shiitake mushroom flour led to a significant decrease in the whiteness index of the baked bread. Similar downward trends in WI were also observed in earlier work by Kobus et al.^17^, which examined the effect of chaga addition on the color characteristics of gluten-free bread. Increasing the proportion of chaga resulted in a marked reduction in lightness and WI, suggesting a comparable mechanism by which intensely colored mushroom materials influence bread color.

In the present study, the addition of maitake to gluten-free bread led to a significant increase in the browning index (BI). This parameter rose from 23.52 in the control to 40.78 in bread containing 20% maitake, indicating intensified browning reactions during baking. Nevertheless, based on ANOVA and Tukey’s test (*p* > 0.05), no significant differences in BI were found between the control and bread enriched with 5% maitake, suggesting that the effect becomes more pronounced only at higher addition levels. Similar relationships were reported by Kobus et al.^17^ for chaga-enriched gluten-free bread: BI increased relative to the control, although raising the chaga level from 5% to 10% and 15% did not produce further significant differences in BI, implying that bread darkening may reach a plateau at moderate addition levels. Other studies^33,40^ corroborate this trend—breads enriched with powdered mushrooms exhibit lower lightness and higher browning index values, resulting in a darker final product.

### Textural properties of bread

The results of textural properties are presented in Table 4.

**Table 4:** Textural properties of breads.

Probe	Hardness [*N*]	Elasticity [-]	Cohesiveness [-]	Chewiness [*N*]
**0**	17.62 ± 0.97^bc^	0.925 ± 0.026^ab^	0.465 ± 0.030^a^	7.58 ± 0.81^a^
**5**	17.10 ± 0.72^c^	0.936 ± 0.024^a^	0.477 ± 0.002^a^	7.62 ± 0.25^a^
**10**	18.58 ± 0.54^abc^	0.885 ± 0.020^bc^	0.484 ± 0.029^a^	7.98 ± 0.70^a^
**15**	19.28 ± 1.78^ab^	0.868 ± 0.041^bc^	0.470 ± 0.025^a^	7.87 ± 0.98^a^
**20**	19.30 ± 0.91^a^	0.822 ± 0.054^c^	0.468 ± 0.016^a^	7.42 ± 0.53^a^

0 – control probe. 5 – gluten-free bread with the addition of 5% maitake mushroom; 10 – gluten-free bread with the addition of 10% maitake mushroom; 15 – gluten-free bread with the addition of 15% maitake mushroom; 20– gluten-free bread with the addition of 20% maitake mushroom. Data value of each parameter with different superscript letter in rows are significantly different (Tukey test. p *≤* 0.05).

Statistical analysis confirmed significant changes in bread hardness across formulations with different levels of maitake mushroom. It should be noted, however, that for breads containing up to 15% addition, hardness did not differ significantly from the control. A significant increase in hardness was observed only when the maitake level reached 20%. Moreover, the lowest crumb hardness (17.10 N) occurred at 5% maitake and differed significantly from the 15% and 20% variants. In the study by Lu et al.^39^, crumb hardness likewise decreased upon introducing small amounts of dried mushrooms; the authors observed this trend for breads supplemented with porcini and white button mushrooms. Notably, that experiment evaluated textural parameters in breads fortified with various mushrooms at 5–15%, and the textural outcomes differed significantly depending on mushroom species. In breads fortified with 5% and 10% porcini, crumb hardness decreased significantly; only at 15% did hardness approach the control. By contrast, shiitake initially reduced hardness, but at a 15% level the bread’s hardness more than doubled. Angioloni^45^ reported that the harder the crumb, the lower the consumer ratings. Thus, maintaining hardness close to the control may be critical for consumer acceptance.

The greatest springiness—reflecting the crumb’s ability to recover its shape after deformation—was observed in the control. As the maitake level increased, springiness tended to decline, though not always significantly. Crumb springiness did not change significantly relative to the control up to a 10% maitake addition; increasing the level to 15% caused a significant decrease. A further increase to 20% did not yield additional statistically significant changes, although mean values continued to trend downward. The inclusion of functional additives—such as dried mushrooms—into wheat or gluten-free breads most often reduces springiness^20,30,44,46^, although some reports indicate that these changes may remain nonsignificant at lower mushroom levels, consistent with our findings. Sławińska et al.^33^ analyzed the quality of wheat bread with white and brown *Agaricus bisporus*. They found that springiness declined significantly even at a 2.5% addition of white *A. bisporus*, whereas fortification up to 5% with brown *A. bisporus* did not significantly affect springiness. Reduced springiness (i.e., a less elastic crumb) may be perceived negatively by consumers. Norton^47^ noted that instrumentally measured springiness of white bread correlated positively with panelists’ perceived springiness; thus, this parameter may serve as a useful predictor of perceived bread freshness.

Cohesiveness describes the extent to which a material can be deformed before it fractures^48^. High cohesiveness prevents disintegration during mastication, whereas too little leads to crumbling^49^. Mean cohesiveness values ranged from 0.465 (control) to 0.484 (15% sample). However, statistical analysis did not confirm the significance of these changes. Łysakowska et al.^46^ and Ulziijargal et al.^30^, who added various mushroom powders to wheat bread formulations, likewise observed no significant changes in cohesiveness—but only for breads containing no more than 5% and 9% of the tested material, respectively. In Łysakowska et al.^46^, increasing Lion’s Mane to 12% caused a significant decrease in cohesiveness. Interestingly, Takeno et al.^50^ reported that, in wheat bread with up to 30% fresh mushrooms, cohesiveness increased with higher levels of Enokitake, but decreased at higher levels of Shiitake.

Chewiness increased slightly as maitake was raised from 0 to 15%. In turn, for breads with a higher maitake level, a decrease in crumb chewiness was recorded. The statistical analysis did not confirm the significance of these changes. It is worth noting that chewiness—the work required to chew a food bite—depends on other textural parameters such as hardness, springiness, and cohesiveness^48^. Consequently, changes in chewiness can be difficult to predict. It is likely that increasing hardness, together with decreasing springiness and relatively stable cohesiveness, helped maintain crumb chewiness at a level close to that of the control.

### Content of macro- and microelements

The results of the micro- and macroelement analysis of gluten-free bread supplemented with maitake mushroom are presented in Table 5.

**Table 5:** Elemental compositions of bread.

Probe	Macroelements [mg⋅kg^-1^d. w.]					Microelements [mg⋅kg^-1^ d. w.]	
	K	*P*	Mg	Ca		Fe	Zn
**0**	2608.6 ± 20.0^e^	2256.1 ± 124.3^d^	766.2 ± 15.9^b^	268.3 ± 11.8^bc^	23.2 ± 0.4^c^		17.1 ± 0.4^e^
**5**	4217.4 ± 35.2^d^	2586.6 ± 12.6^cd^	775.2 ± 11.5^b^	330.1 ± 13.6^a^	26.1 ± 0.4^c^		24.8 ± 1.1^d^
**10**	5084.9 ± 34.4^c^	2968.7 ± 18.7^c^	834.3 ± 41.8^ab^	316.0 ± 16.0^ab^	28.6 ± 0.9^b^		35.9 ± 1.1^c^
**15**	7255.4 ± 130.2^b^	3460.0 ± 50.3^b^	874.4 ± 20.4^a^	316.7 ± 16.5^ab^	33.5 ± 0.7^b^		42.9 ± 2.0^b^
**20**	8275.0 ± 369.1^a^	4013.0 ± 319.1^a^	881.5 ± 35.6^a^	259.4 ± 31.5^b^	36.1 ± 0.4^a^		51.4 ± 6.2^a^

0 – control probe. 5 – gluten-free bread with the addition of 5% maitake mushroom; 10 – gluten-free bread with the addition of 10% maitake mushroom; 15 – gluten-free bread with the addition of 15% maitake mushroom; 20– gluten-free bread with the addition of 20% maitake mushroom. Data value of each parameter with different superscript letter in rows are significantly different (Tukey test. p *≤* 0.05).

The contents of K, P, Mg, Fe, and Zn increased with higher levels of maitake. For potassium and zinc, these changes were already significant at a 5% addition (*p* < 0.05). In bread with 20% maitake, the concentrations of these elements were more than threefold relative to the control. Similar trends have been reported elsewhere^20,46^. An exception was noted for phosphorus and potassium in studies with other mushrooms: when Reishi fruiting bodies or Lion’s Mane powders replaced part of the flour, P and K decreased with increasing mushroom level, which the authors attributed to higher P and K contents in type 750 wheat flour compared with those particular mushrooms.

In the study by Niedzielski et al.^51^, the mineral composition of 12 mushroom species—including maitake (*Grifola frondosa*)—was analyzed. Maitake exhibited the highest contents among all samples for calcium (2,481 mg·kg^-^¹ _**d.m.**_), magnesium (1,453 mg·kg^-^¹ _**d.m.**_), iron (214 mg·kg^-^¹ _**d.m.**_), manganese (32 mg·kg^-^¹ _**d.m.**_), and zinc (246 mg·kg^-^¹ _**d.m.**_). Notably, very high levels of potassium (31,940 mg·kg^-^¹ _**d.m.**_), phosphorus (26,910 mg·kg^-^¹ _**d.m.**_), and sodium (153 mg·kg^-^¹ _**d.m.**_) were also reported relative to other mushrooms. This rich mineral profile makes maitake not only a valuable food raw material but also an attractive ingredient for the pharmaceutical sector^52,53^.

### FTIR spectroscopic analysis

**Fig. 2:**
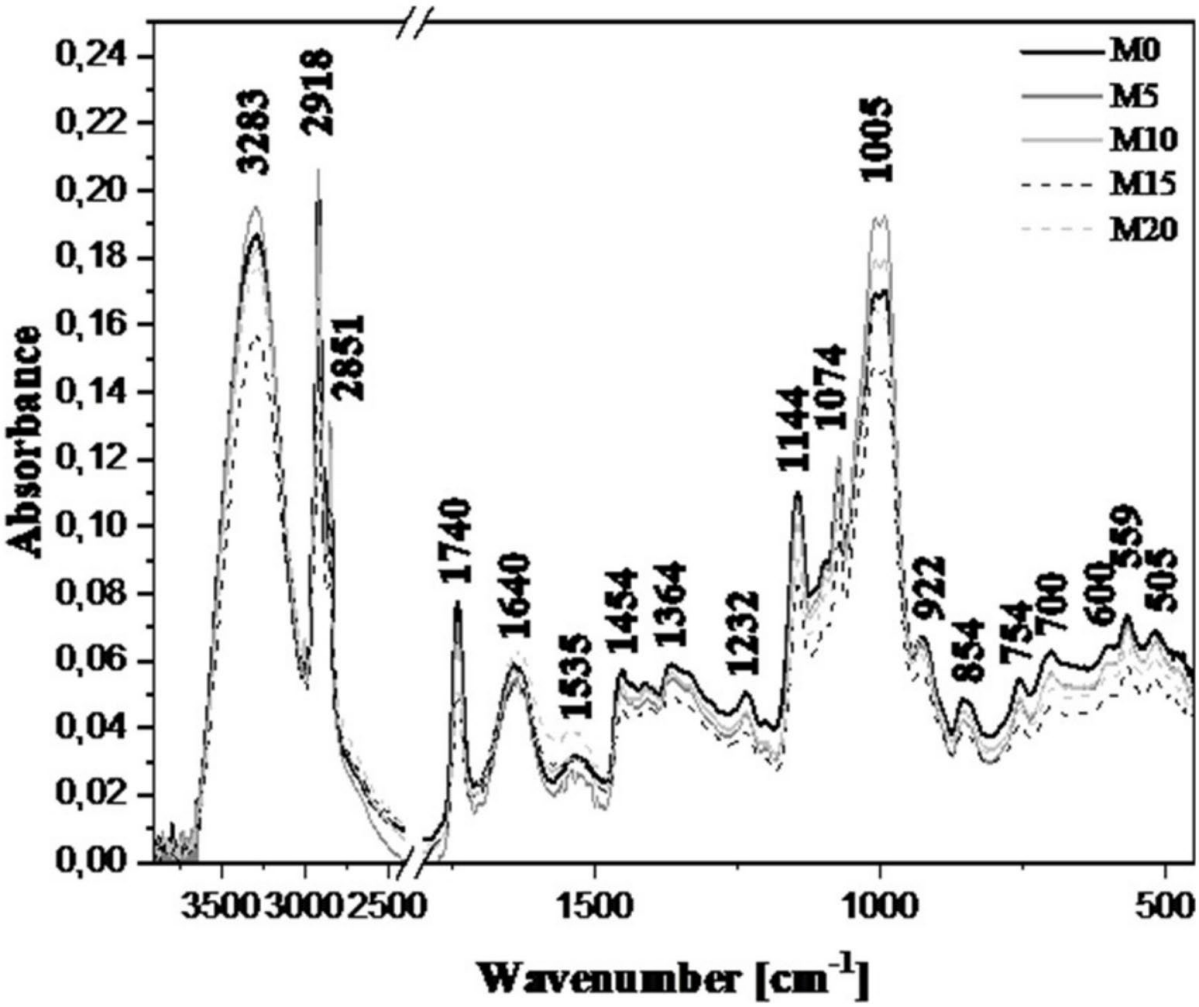
The location of the maxima of absorption bands FTIR with arrangement of appropriate vibration for selected for sampling made in terms of spectra range 3900–450 cm ^-1^*.* Continuous black line - control bread without additives, graphite line - bread with 5% addition of maitake mushrooms, light gray line - bread with 10% addition of maitake mushrooms, dashed black line - bread with 15% addition, dashed gray line − 20% addition of maitake mushrooms.

For a detailed, molecular-level characterization of breads enriched with maitake mushroom, FTIR spectroscopy was employed. To facilitate interpretation and comparison, all spectra are compiled in Fig. 2, and the band assignments are summarized in Table 6.

The first, very intense and broad band with a maximum at 3283 cm^-¹^ corresponds to the typical O–H stretching vibrations. The high intensity of this band arises from the large number of –OH groups present both in the basic bread constituents and in the added ingredients, and likely also from water molecules in the samples. Depending on the amount of the additive, the signal intensity varies^29,54^. In general (apart from the 5% maitake addition), the intensity of this band decreases, which may result from changes in the amount of bound water or in the number of hydroxyl groups accessible within the bread structure. Importantly, the increasing incorporation of maitake (up to 20%) led to a significant increase in bread moisture (from 49.38% to 53.12%, see Table 1). The shifts and changes in the intensity of this hydroxyl band region are consistent with interactions between fungal polysaccharides and starch/protein components between the fungal polysaccharides and starch/protein components, leading to increased water retention and stabilizing the bread structure, as evidenced by the higher final moisture content.

The next two bands, with maxima at 2918 cm^-^¹ and 2851 cm^-^¹, are assigned to the C–H stretching vibrations in –CH_2_ groups^29,54^. The change in this range is very significant, as it reflects the increased content of bond-rich components, particularly lipids (Table 2) and polysaccharides/structural fiber, contributed by the mushroom powder. The significant increase in fat content (from 5.40% in the control group to 6.27% with 20% addition, Table 2) confirms the observed changes in these hydrophobic regions, suggesting the incorporation of mushroom-derived components into the bread matrix structure.

The band with a maximum at 1740 cm^-^¹ originates from carbonyl group vibrations. It is most intense in bread without the mushroom addition; for samples containing 5% and 10% maitake, the intensity decreases progressively. For the 15% and 20% additions, the signal remains at a similar level, though lower than at 10%. This band is characteristic of various lipid fractions (e.g., lipids and, in this type of sample, fats). The bands in the region are critical for evaluating protein structure. Amide I band (around 1643), mainly related to stretching vibrations, and Amide II band (around 1530), associated with bending and stretching, showed clear variations with maitake inclusion. The overall increased intensity and subtle shifts in these amide bands are directly linked to the higher protein content of the fortified breads (Table 2), which rose from 7.18% in the control to 9.77% at 20% maitake addition. These changes suggest that the addition of maitake proteins (which differ in structure from the rice and corn proteins) impacts the overall protein network architecture and its interaction with the starch phase, potentially contributing to the observed changes in texture and specific volume (Table 1)^29,54^.

In summary, the characteristic vibrations in the 1800–1500 cm^-^¹ region point to interactions between maitake mushroom constituents and the proteins within the bread matrix.

It is also important to underscore differences in band intensities between 1460 and 1200 cm^-^¹, a region associated with lipid fractions; note that its intensity decreases markedly upon addition of mushrooms. The apparent reduction in intensity in these regions may stem from shifts in the relative proportions of lipid, polysaccharide, and protein components.

The signal with a maximum at 1451 cm^-^¹ is characteristic on the one hand of protein structures^54,55^, but also of –CH_2_ bending and *ν*(–COO) vibrations. The band with a maximum at 1232 cm^-^¹ is assigned to Amide III, likewise characteristic of protein structures (including C–N and C–C stretching and N–H bending).

Bands located at 1144, 1074, 1005, and 922 cm^-^¹, corresponding to C–O, C–C, and C–O–C stretching vibrations^29,54^, also play an essential role in interpreting the spectrum; their intensity may increase in the presence of carbohydrate additives. The FTIR analysis confirms modifications of the starch structure in both crystalline and amorphous domains, which aligns with the observed significant rise in bread hardness only at 20% inclusion and the decrease in crumb springiness, suggesting a structural stiffening of the crumb matrix due to maitake-starch interactions. This region reflects, among other features, the fiber content of the samples, and a pronounced effect of the additive is apparent. The profile is rather irregular and warrants further study. Nonetheless, the high intensity of these bands is consistent with the high energy value of the product.

Finally, it should be noted that significant changes are also observed below 900 cm^-^¹. This region is mainly associated with vibrations within bonds of the polysaccharide fractions that constitute the core structure of the samples^27,29,54^. Bands in this range correspond in particular to *α*−1,4- and *α*−1,6-glycosidic linkages that connect the monomeric units of starch^29,54,56^. The changes in the intensity of these signals, evident at different maitake inclusion levels, indicate modifications of the starch structure in both crystalline and amorphous domains.

**Table 6:** The location of the maxima of absorption bands FTIR with arrangement of appropriate vibration for selected for sampling made in terms of spectra range 3700–490 cm^*−* 1^.

Maximum Position (cm^−1^) All samples	Types and Origin of Vibrations
3283	*ν*_st_.(—OH) and intermoleculary H-bonded
2918	*ν*_st_. (C—H) in CH_2_
1740	*ν* (C=O)
1640	*δ* (O—H) and Amide I
1535	Amide II
1454	*ν* (C—C)
1364	*δ* (–CH_2_) + *ν* (COO)
1232	Amide III
1144	*ν*_m_ (C—O) and *ν*_m_ (C—C)
1074	*ν* (C—O—C)
1005	*ν* (C=C) and skeletal vibrations in the pyranose ring
922	vibrations in *α*−1,4-glycosidic
854	*α*−1,6-glycosidic bonds in starch structure

### Consumers’ bread preferences

**Fig. 3:**
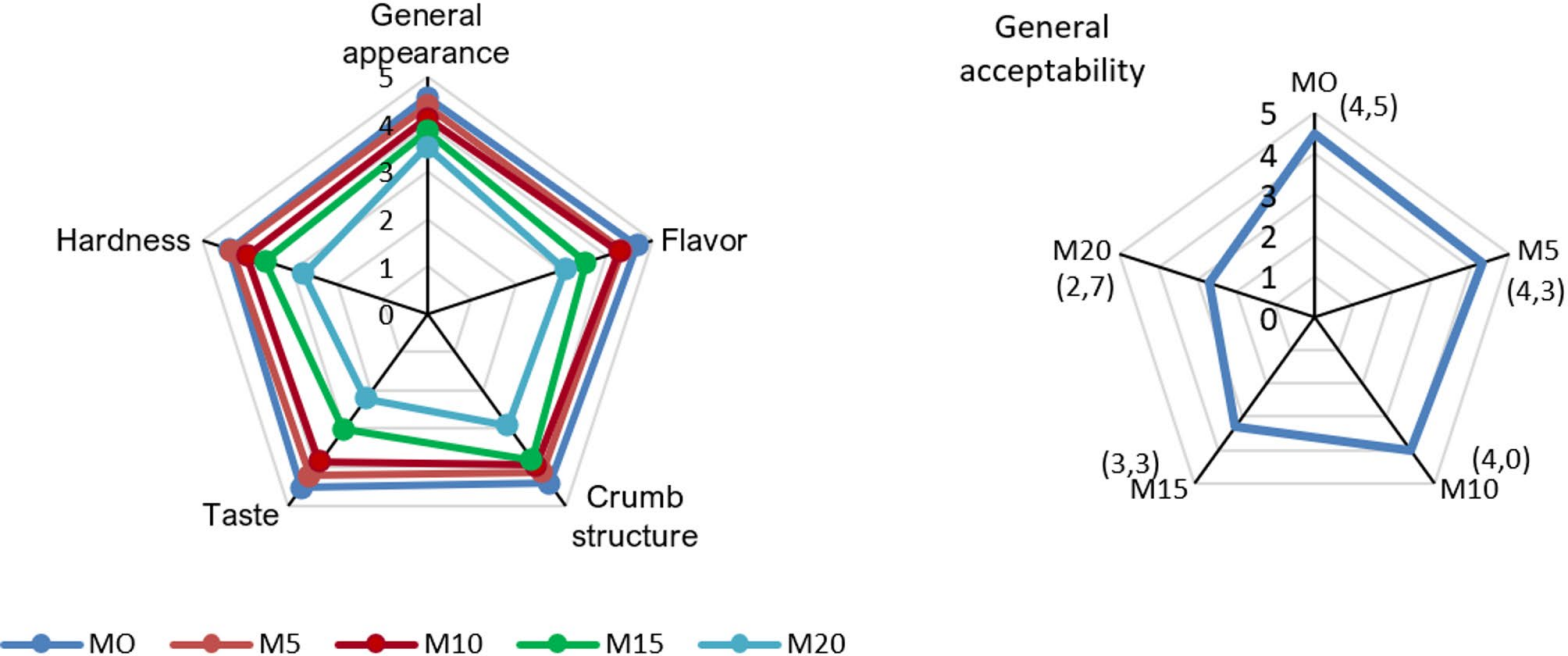
Consumer preference ratings for bread characteristics: M0 — control sample; M5 — gluten-free bread with 5% maitake; M10 — gluten-free bread with 10% maitake; M15 — gluten-free bread with 15% maitake; M20 — gluten-free bread with 20% maitake.

Dietary preferences for bread are driven primarily by sensory attributes (taste, aroma, texture, appearance), but also by nutritional value (e.g., fiber, vitamins, minerals) and convenience. Figure 3 presents the results of the acceptability assessment of breads enriched with maitake (*Grifola frondosa*). Consumer acceptability testing enables analysis of preferences and is a fundamental tool when introducing new products to the market. In line with current trends toward innovative ingredients, consumers increasingly consider breads formulated with mushrooms rich in bioactive compounds. The sensory attributes were evaluated on a 5-point hedonic scale (appearance, taste, aroma, texture, overall acceptability).

Bread with mushroom addition was considered acceptable when the mean overall acceptability score was *≥* 3. The highest acceptability—besides the control sample (M0; 4.5 points, without mushrooms)—was recorded for bread with a 5% mushroom addition (M5; 4.3 points). The most highly rated features were external appearance, aroma, taste, and texture. As the level of maitake (*Grifola frondosa)* increased, panelists assigned progressively lower scores for individual sensory attributes. Bread containing 20% maitake was judged least palatable (2.2 points) and exhibited inadequate structure and texture (2.9 and 2.7 points, respectively). The low score likely resulted from a bitter aftertaste, which also reduced the overall acceptability rating to 2.7 points.

Comparable findings were reported in a sensory analysis of gluten-free bread enriched with chaga (*Inonotus obliquus*) powder, where consumers most preferred samples with low inclusion levels (up to 10%). Higher concentrations (15% and 20%) markedly decreased sensory scores—particularly for taste, texture, and overall acceptance—attributed to the intense flavor and very dark color of the bread^17^.

Similarly, Łysakowska et al.^20^ showed that bread supplemented with Ganoderma lucidum (Curtis) P. Karst. (*Reishi mushroom*) powder at 3–6% did not differ from the control in appearance, aroma, taste, and elasticity, whereas higher levels (9–12%) caused a pronounced deterioration in taste and aroma, suggesting that the intense aroma and flavor of mushrooms may limit consumer acceptance. Likewise, studies by Gaglio et al.^57^ found that adding *Pleurotus eryngii* powder intensified crust and crumb color and affected aroma and taste, while negatively influencing crumb porosity and elasticity. Nevertheless, the overall sensory evaluation of bread containing up to 10% *P. eryngii* powder indicated an acceptable sensory profile^57^. These results suggest that selected mushrooms have considerable potential as functional bread additives. When used at lower inclusion levels, they do not substantially impair sensory qualities while providing a source of bioactive compounds. However, careful selection of the mushroom dosage during bread fortification is crucial to maintaining a balance between health benefits and consumer acceptance.

## Materials and methods

### Raw material

The dried maitake (*Grifola frondosa*) purchased for testing have certificate number 33/1610/2023. The raw material was ground by a knife mill and sieved through a stainless steel sieve with a mesh size of 0.2 mm. According to the data on the packaging, maitake mushroom (per 100 g) contains: fat 5.8 g, including saturated fatty acids 1.1 g, carbohydrates 28.3 g, including sugars 4.2 g, fiber 33.7 g, protein 23.3 g. The energy value is 1362 kJ/326 kcal.

### Bread preparation procedure

The maitake-enriched loaves of bread were prepared following the formulations listed in Table 7. The dough was mixed using a laboratory spiral mixer (Kenwood. Havant. UK) for 5 min. Subsequently a 537.5 g portion of the dough was transferred into a loaf pan and allowed to undergo final fermentation for 40 min at 37 °C with 80% relative humidity. The loaves were then baked in a convection-steam oven (Houno. Randers. Denmark) at 230 °C for 40 min. To determine the required dough water, the water absorption index (WAI) of the flours and maitake powder was measured. A suspension was made by mixing 1 gram of the powdered sample with 10 milliliters of distilled water at room temperature for 30 min. with gentle stirring throughout. The mixture was then centrifuged at 3000 r/m for 15 min. After carefully pouring off the supernatant, the remaining solid was weighed. The WAI was calculated as the weight of the resulting gel divided by the original weight of the powdered sample^58^. It was 500, 503, 516, 526 and 536 g for control probe and for gluten-free bread with the addition of 5%. 10%. 15% and 20% respectively. Percentages in the formulations follow the baker’s percentage convention, in which each ingredient is expressed as a percentage of the total flour (all flour types combined), which is set to 100%.

**Table 7:** The composition of bread dough with maitake addition.

Probe code	0	5	10	15	10
*Ingredients*	[%]				
Riceflour	50	45	40	35	30
Cornflour	40	40	40	40	40
Potatostarch	10	10	10	10	10
Water	100	100	100	100	100
Rapeseedoil	6	6	6	6	6
Dryyeast	1.4	1.4	1.4	1.4	1.4
Salt	2.4	2.4	2.4	2.4	2.4
Sugar	2	2	2	2	2
Groundflaxseeds	3	3	3	3	3
Maitake	0	5	10	15	20

0 – control probe. 5 – gluten-free bread with the addition of 5% maitake mushroom; 10 – gluten-free bread with the addition of 10% maitake mushroom; 15 – gluten-free bread with the addition of 15% maitake mushroom; 20– gluten-free bread with the addition of 20% maitake mushroom.

### Chemical analysis

#### Extracts preparation

For the extracts preparations, the mass of 1 g for dried maitake and 2 g for bread was weighted and contacted with 30 mL of distilled water. The mixture was mixed for 1 h using magnetic stirrer (100 r/m, 25 °C), and then preserved at room temperature in darkness for 24 h. Afterwards, the extracts were gathered to the vessel, and 30 mL of methanol was added to the remaining raw material. As previously, the mixture was stirred for 1 h and maintained at room condition without access to light for 24 h. Finally, these two fractions were mixed together in a *v/v* = 1:1.

#### Total phenolic and flavonoid content and antioxidant activity analysis

The Total Phenolic Content (TPC), Total Flavonoids Content (TFC), and the 2,2-diphenyl-1-picrylhydrazyl (DPPH) and the Ferric Reducing Antioxidant Power (FRAP) antioxidant activity tests were performed spectrophotometrically on UV-Vis 1800 spectrophotometer (Shimadzu, Kyoto, Japan). The TPC was measured following the procedure outlined by Pecyna et al. The extracts were contacted with 10% Na_2_CO_3_and Folin-Ciocalteu solutions. The TFC was evaluated according to the methodology presented by Kobus et al^59^. To determine TFC, the extracts were contacted with 2% methanol solution of AlCl_3_ × 6 H_2_O. The prepared mixtures were maintained in darkness before measurements for 30–10 min, respectively. The TPC and TFC values were expressed as mg GAE or QE·g_d.m._^−1^. The DPPH and FRAP activity studies were conducted according to the methodology described by Kobus et al. The extracts were contacted with 5 mL DPPH or FRAP solutions and left in darkness for 30–10 min, respectively. In both cases, the antioxidant activities were expressed as µg TE·g_d.m_^−1^^59,60^.

#### Determination of protein content

Protein content was determined by the Kjeldahl method in accordance with ISO 1871:2009. The method involves converting organic nitrogen compounds to ammonium sulfate by digestion with concentrated sulfuric acid in the presence of a catalyst, alkalizing the digest, distilling the released NH_3_, and titrating the ammonia captured in boric acid with hydrochloric acid.

#### Determination of fat content

Fat content was determined using a Soxtec 8000 apparatus (application ASN 310). The Soxhlet method consists of repeated continuous extraction of lipids from a pre-dried, comminuted sample with an organic solvent, removal of the solvent, and gravimetric determination of the extracted lipid fraction. The analysis was carried out in accordance with ISO 659:1998 (Geneva, Switzerland).

### Spectroscopic measurements (FTIR)

Infrared spectra (FTIR) were recorded using an IRSpirit spectrometer equipped with an ATR module (SHIMADZU) featuring a 45° ZnSe crystal enabling multiple internal reflections. Samples were prepared according to the procedure described in Sect. 3.2, and 40 scans were acquired for each sample over the 450–3900 cm^-^¹ range. The resulting spectra were averaged. Crystal cleanliness (ZnSe) was maintained by using ultrapure solvents from Merck KGaA. Spectral analysis was performed with Grams/AI software (version 9.3, ThermoGalactic Industries, Waltham, MA, USA) and OriginPro 2021b (version SR1 9.8.5.204, OriginLab Corporation, Northampton, MA, USA).

### Determination of textural properties of bread

Textural parameters were determined 4 h after baking. For sample preparation, loaves were sliced into 10-mm-thick slices; end slices were discarded. Cuboids measuring 30 × 30 × 10 mm were then cut from the central portion of the slices. The samples thus prepared were subjected to a double-compression test (TPA, Texture Profile Analysis). Measurements were performed using a universal testing machine (Zwick/Roell, Z0.5, AG, Ulm, Germany). Each sample was compressed to 50% of its original height at a constant crosshead speed of 50 mm·min^-^¹. A 500 N measuring head and a flat, cylindrical probe with a diameter of 100 mm were used. The measurements were carried out in constant laboratory conditions, at temperature of 25 °C. From the resulting force–deformation curves, the following properties were calculated with testXpert II software: hardness [N] (maximum force during the first compression), springiness [–] (ratio of deformation during the second to the first compression cycle), cohesiveness [–] (ratio of the area under the force–time curve in the second to the first compression cycle), and chewiness [N] (product of hardness, springiness, and cohesiveness)^61^. Measurements were performed in five replicates.

### Colour analysis

Colour measurements were carried out using a 3Color^®^ SF80 spectrophotometer. Each sample was analyzed ten times (light source: D65. observer angle: 10°. measurement aperture: 8 mm). Results were reported in the CIELab colour space. with the following parameters recorded: L* (lightness). a* (green to red shift). and b* (blue to yellow shift). Higher a* and b* values indicate greater intensities of red and yellow tones. respectively.

Additionally colour saturation C* was calculated using the formula (1):

1 $$\:{C}^{*}=\sqrt{{\mathrm{a}}^{*2}+{\mathrm{b}}^{*2}}$$

Moreover. the overall colour difference (∆E) between the control gluten-free bread and the maitake-supplemented bread was calculated using the following Eq. (2):

2 $$\:\varDelta\:E=\sqrt{{(\varDelta\:{L}^{*})}^{2}+{{(\varDelta\:{a}^{*})}^{2}+(\varDelta\:{b}^{*})}^{2}}$$

Where: ∆*L*, ∆*a* and ∆*b*—indices of the difference in the colour of the surfaces of samples compared with the control bread [40]. The Whiteness Index (WI) was determined using the following formula (3):

3 $$\:WI=100-\sqrt{{(100-{L}^{\mathrm{*}})}^{2}+{{a}^{*}}^{2}+{{b}^{*}}^{2}}$$

The Yellowness Index (YI) was determined using the following formula (4):

4 $$\:YI=142.83\bullet\:\frac{{b}^{\mathrm{*}}}{{L}^{\mathrm{*}}}$$

The browning index (BI) was determined using the following formula (5):

5 $$\:BI=\frac{100(x-0.31)}{0.17}x$$

Where x was calculated using Eq. (6):

6 $$x=\:\frac{({a}^{\mathrm{*}}+1.75{L}^{\mathrm{*}})}{(5.645{L}^{\mathrm{*}}+{a}^{\mathrm{*}}-3.012{b}^{\mathrm{*}})}$$

WI is the whiteness index. YI is the yellowness index^62^ and BI^63^ is the browning index.

### Determination of element content

The ground bread, weighing 5 g, was pressed using a hydraulic press (Fuji, model 181–1375, Madison, USA). The elemental composition of the samples was determined using an EDX-8100P X-ray dispersive fluorescence spectrometer (Shimadzu, Kyoto, Japan). The test was repeated three times^64^.

### Determination of sensory parameters of bread

The bread evaluation was conducted using a Likert scale to examine the degree of ordering and to identify attributes influencing consumer acceptance. The study involved 20 panelists as potential consumers (45% women, 55% men; 30–50 years old). All panelists declared that they regularly consume bread and provided informed consent to participate. Prior to testing, loaves were sliced into 1-cm pieces containing both crumb and crust. Samples were randomly coded. Panelists assessed: general appearance (shape, crust colour, baking uniformity), flavor (freshness, intensity, pleasant scent), crumb structure (porosity, elasticity, moisture), taste (intensity, naturalness of taste), hardness (crispness of the crust, porosity), and overall acceptability. A 5-point hedonic scale was used, where 1 = definitely does not meet expectations, 2 = rather does not meet expectations, 3 = neither meets nor fails to meet expectations (neutral), 4 = rather meets expectations, and 5 = definitely meets expectations. Scores of 2 and below indicated product rejection by panelists, whereas scores of 3 and above indicated product acceptance.

### Statistical analysis

The obtained results were subjected to analysis of variance (ANOVA). The significance of the variations among the mean values was assessed using Tukey’s test at a significance level of *p* < 0.05. All statistical data treatments were performed with Statistica software (Statistica 13; StatSoft Inc. Tulsa. OK. USA).

## Conclusions

Maitake (*Grifola frondosa*) proved to be a promising functional fortifier for gluten-free bread, delivering substantial gains in phenolics, flavonoids, and antioxidant capacity while enriching the product with key minerals (K, P, Mg, Fe, Zn). Technologically, maitake increased bread yield and moisture and tended to lower total baking loss; textural performance remained broadly comparable to the control up to about 10–15% inclusion, with a significant rise in hardness only at 20%. Colour shifted in a dose-dependent manner—crumbs darkened (lower L*) and redness increased (higher a*), whereas b* and chroma showed no significant changes; ∆E values above 5 indicated perceptible differences, alongside higher YI, a non-significant downward trend in WI, and elevated BI at higher additions. The FTIR analysis clearly confirmed the above results and provided evidence that the addition of maitake mushrooms leads to profound molecular modifications of the bread matrix, encompassing polysaccharide, protein, and lipid fractions. Changes in the intensity and positions of bands characteristic of hydroxyl groups indicate strong interactions between fungal polysaccharides and starch and proteins, which translates into increased water-binding capacity and stabilization of the crumb structure. Taken together, the FTIR results provide compelling evidence that the addition of maitake does not simply enhance the chemical composition of bread but leads to a comprehensive remodeling of its molecular structure. These changes may have crucial implications for the technological, textural, and potentially functional properties of bread, confirming its high potential as an ingredient in a new generation of bakery products with enhanced nutritional value. In terms of consumer acceptability, the highest scores were obtained for breads containing 5–10% of maitake, whereas a 20% mushroom addition resulted in a decrease in sensory quality due to increased bitterness and poorer texture. Overall, maitake enables the development of gluten-free breads with a cleaner label and high nutrient content. However, careful selection of the mushroom dosage during bread fortification is crucial to maintaining a balance between health benefits and consumer acceptance.

## Data Availability

All data generated or analysed during this study are included in this published article.
